# An introduction to polygenic scores – methodological basics and recent advances

**DOI:** 10.1515/medgen-2026-3009

**Published:** 2026-07-08

**Authors:** Hannah Klinkhammer, Andreas Mayr, Carlo Maj

**Affiliations:** Marburg University Institute for Medical Biometry and Statistics Hans-Meerwein-Str. 6 35032 Marburg Germany; Marburg University Institute for Medical Biometry and Statistics Hans-Meerwein-Str. 6 35032 Marburg Germany; Marburg University Center for Human Genetics Baldingerstr. 35043 Marburg Germany

## Abstract

Polygenic scores (PGS) allow the estimation of genetic predisposition to complex diseases and traits. Based on results from genome-wide association studies (GWAS) and data from large deeply phenotyped population biobanks, numerous PGS have been developed in recent years. These scores summarize the combined effects of many genetic variants and can support risk stratification for multifactorial diseases based on an individual’s genetic susceptibility. In this review, we introduce the basic principles and methods of PGS and explain how they are generated and applied. We outline their potential translational role, particularly in the context of precision medicine and personalized risk stratification for preventive measures, in combination with established clinical risk factors. At the same time, we discuss important limitations, including limited generalizability across populations and the issue of missing heritability. Finally, we highlight current methodological developments and future perspectives for the integration of PGS into clinical practice.

## Introduction

In the past few decades, genome-wide association studies (GWAS) have established the standard analytical framework for identifying common genetic variants associated with complex traits and diseases [1, 2]. Large-scale GWAS have identified thousands of associated loci across the human genome, demonstrating that the genetic architecture of most complex phenotypes is highly polygenic, with numerous variants of small individual effects contributing to the overall genetic liability [3].

This progress has been enabled by major advances in genotyping and sequencing technologies [4, 5]. The decreasing cost of high-density single nucleotide polymorphism (SNP) arrays has facilitated the collection and analysis of very large population cohorts, allowing systematic assessment of common variant effects [6, 7]. More recently, improvements in sequencing and the development of large-scale biobanks and international consortia have enabled genome-wide data generation at unprecedented scale and cost-efficiency [8–12]. As a result, genetic association studies have expanded from analyses involving a few hundred individuals to those including millions, achieving the statistical power necessary to detect associations with increasingly modest effect sizes [8].

The initial objective of GWAS was the identification of genetic loci associated with disease risk to provide mechanistic insight and inform potential therapeutic targets [1]. This approach has been successful in mapping reproducible associations and revealing biological pathways relevant to human traits and diseases [13]. However, an important limitation soon became evident: the identification of a risk locus does not necessarily translate into predictive utility at the individual level. For most complex traits, the effect size of a single common variant is small, and its contribution to disease risk is very small when considered in isolation [1]. A few notable exceptions exist, including the APOE ε4 allele in Alzheimer’s disease, variants in ™TO associated with obesity, and common missense and loss-of-function variants in PCSK9 affecting lipid levels, which show detectable phenotypic differences among individuals carrying different genotypes [14 – 17]. Another well-known example of a comparatively oligogenic architecture is represented by autoimmune diseases, in which variants within the HLA region can explain a substantial proportion of SNP-based heritability [18]. Nevertheless, such examples are rare, and for most phenotypes genetic susceptibility arises from the combined influence of many small-effect variants [2, 3].

This recognition has led to the development of polygenic scores (PGS), also known as polygenic risk scores (PRS) when designed to estimate susceptibility to disease [19, 20]. A PGS aggregates the effects of multiple genetic variants into a single quantitative measure of genetic liability, typically computed as a weighted sum of allele dosages using GWAS-derived effect estimates [21]. The availability of large-scale biobank datasets and high-dimensional genotype–phenotype data has further enabled the development of improved prediction models, including those based on direct genotype-to-phenotype regression, which better approximate the true distribution of genetic effects [22 – 24].

The objective of this review is to provide a concise methodological and conceptual overview of PGS; from their derivation and statistical assumptions to their current limitations and emerging translational applications. We describe the main methodological approaches for PGS estimation and interpretation, outline the major sources of publicly available PGS repositories, refer to currently implemented models in clinical practice, and discuss key challenges and future directions for their robust and generalizable implementation in translational research and clinical applications.

## Methods for PGS development

In order to compute PGS, a structured approach for combining the small effects of many genetic variants into a single measure is needed. As the goal is prediction, a PGS could be generally derived via a multivariable regression model and correspond to a structured linear predictor

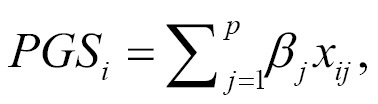



where the PGS of person *i* is derived as the weighted sum of the number of risk alleles of variants *j* = 1, … *p* (*x_ij_*) with effect estimates *β_j_*. However, this is statistically and computationally challenging because most traits are influenced by a very large number of variants and because variants in close proximity in the genome are correlated through linkage disequilibrium (LD). Therefore, most commonly used PGS methods rely on combining GWAS summary statistics (e.g., *β_j_* that have been estimated separately) and adjust for LD structure to derive an individual-level genetic score in an independent target cohort with genotype data. As such, development of a PGS with these methods involves many statistical estimates (GWAS, LD matrix, PGS effect estimates) and is sensitive to high quality data. Therefore, it is crucial to rely on well powered GWAS that incorporate rigorous quality controls and take further adjustments, in particular for genetic ancestry, into account. Beyond summary-statistics-based approaches, PGS can be derived using individual-level training in large biobank datasets, leveraging joint modeling of variants while accounting for LD within the training cohort.

**Figure 1:**
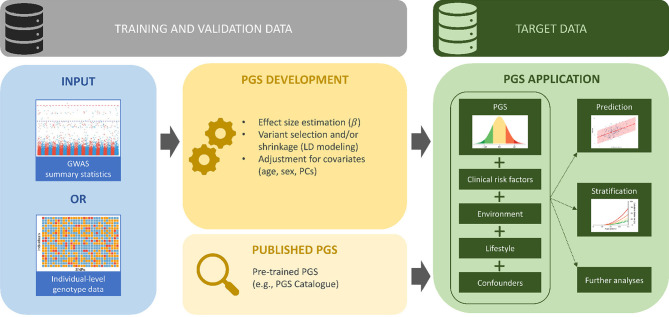
PGS workflow Overview of the end-to-end workflow for polygenic score (PGS) construction and use. PGS models can be developed using training/validation data either from GWAS summary statistics or from individual-level genotype–phenotype data, with key steps including effect size estimation, variant selection/shrinkage (often accounting for LD), and optional adjustment for covariates. Alternatively, previously published scores (e.g., from the PGS Catalog) can be selected and reused. The resulting PGS is then computed in an independent target cohort and integrated with clinical risk factors, environmental and lifestyle variables, and other confounders for prediction, risk stratification, and downstream analyses.

During the last decade, a wide range of PGS methods have emerged spanning from simple clumping and thresholding approaches (PRSice, [25]) to highly complex deep convolutional networks [26]. Methods can be generally categorized into two groups: GWAS-based and individual-level data based approaches (Figure 1) while the majority falls into the first category. Here, PGS are constructed as a weighted sum of effect alleles with weights taken from the estimated effect sizes *β_j_* of the variants in GWAS. One of the first PGS algorithms was clumping and thresholding (implemented in PRSice). To overcome estimation problems due to LD, SNPs are selected for the PGS based on a p-value threshold (thresholding) and a threshold for pairwise correlation within defined windows (clumping). Both thresholds are typically optimized on a validation set or via cross-validation. This simplistic approach neglects a more complex LD structure which led to the development of more advanced algorithms that are based on external population-matched LD reference panels. While lassosum [27] uses penalized regression, most algorithms in this category rely on Bayesian statistics, such as LDpred [28] and PRScs [29]. Those methods differ in the assumed priors and generally include all SNPs in the PGS but modify the effect estimates from GWAS. For example, LDpred assumes a proportion *p* of causal variants and adapts a Gaussian mixture prior, meaning that the effect sizes of the causal variants follow a normal distribution and the effect sizes of the remaining variants are theoretically 0. Using a Bayesian approach, this results in posterior means for the effect size of each variant that are typically different from 0.

Instead of relying on GWAS and subsequently attempting to adjust univariate effect estimates *β_j_* from summary statistics with advanced methods to account for LD, in large cohorts the *β_j_* for PGS can nowadays also be derived directly from individual-level data via multivariable regression models [29]. From a methodological perspective, these methods can be seen as more adequate for the polygenic situation at hand, as they hence account for LD already naturally when estimating the *β_j_* in the first place.

In recent years, statistical learning methods relying on penalized regression via the lasso [22] or statistical boosting [23] have emerged but also Bayesian approaches based on individual-level data have been implemented [31]. Via simultaneous consideration of all SNPs, those methods can yield better PGS estimates and more accurate predictions but rely on individual-level data from large cohorts [23, 32]. In comparison, via GWAS one can easily leverage statistical power by combining results from millions of individuals from multiple different cohorts. The landscape of PGS methods have further been enlarged to multi-PGS approaches (e.g. [33]), multi-ancestry PGS (e.g. [34]) and the integration of a priori knowledge such as functional annotations (e.g. [35]).

The choice of the best method depends on various factors and most importantly on the available data. In the beginnings of PGS, genotype-phenotype associations were particularly available as summary statistics from GWAS which are easily shareable without exposing individual information. More recently, various biobanks were established that employ managed user access to ensure compliance with data sharing agreements.

Among the most prominent, UK Biobank comprises data of approximately half a million individuals [6], however covering mostly European ancestry. Over time, further biobanks were established including FinnGen [36], Biobank Japan [37], deCODE Genetics [38] and the Estonian Biobank [39]. Lastly, data of the All of Us program became available [11] which aims at enrolling at least one million individuals from the US and focuses on building a diverse biobank with respect to historically under-represented groups.

For quantitative traits that are comparatively easy to measure at scale (e.g., blood biomarkers or anthropometric traits), the gain in methodological accuracy enabled by directly modeling variants jointly in large biobanks often translates into improved predictive performance compared with summary-statistics-based approaches. However, for phenotypes that are harder to define reliably in population biobanks – such as neuropsychiatric traits, clinically adjudicated outcomes, or early-onset and rare conditions that are underrepresented in biobanks – PGS construction based on GWAS from deeply phenotyped clinical cohorts assembled by disease-specific consortia is often preferred, as these datasets provide more accurate and harmonized phenotype definitions. Moreover, individual-level data are frequently difficult to share across cohorts due to consent and data-protection constraints, such that GWAS meta-analyses and their summary statistics often represent the only broadly available input for downstream PGS construction.

Beyond the choice between GWAS summary statistics and individual-level training, the optimal PGS model also depends on the underlying genetic architecture. Current frameworks range from (near-)omnigenic/infinitesimal models, which posit that effects are widely distributed across the genome and therefore apply broad shrinkage while retaining a large fraction of SNPs, to sparse models that emphasize variable selection and concentrate weight on a comparatively small set of variants. Sparse approaches tend to perform better when the genetic signal is more concentrated (low-to-moderate polygenicity), whereas highly polygenic or near-omnigenic traits are often captured more accurately by models that assume many non-zero effects. Indeed, some traits are characterized by extremely polygenic architectures, such as height [40], whereas others are more strongly influenced by a small number of loci with larger effects and therefore exhibit a comparatively sparse architecture, for example lipoprotein(a) levels [41], which are largely driven by variation at the LPA locus.

## Availability and downstream application of PGS models

For most PGS methods, the final PGS is a weighted sum of effect alleles, i.e. the model is characterized by the included SNPs, the defined effect allele and the corresponding effect size *β_j_*. Therefore, PGS models are easily shareable and are not subject to privacy concerns. As a result, large databases of published PGS models have formed such as the PGS catalog (https://www.pgscatalog.org/, [42]). Resources like this enable the evaluation of polygenic factors in smaller cohorts without the need to construct a new PGS. Given a target data set for analysis with a specific trait of interest, the choice of a suitable PGS from the PGS catalog depends on different features. One major decision point is the training population of a PGS. In the optimal case, the ancestry of the training population (GWAS and PGS training) match the ancestry of the target data set. Most PGS, however, are derived from individuals of European ancestry and will therefore show strongly decreased performance on individuals of different ancestry. In the PGS catalogue, the ancestry of the training population is indicated for each PGS. Furthermore, it is important to find a PGS with a matching phenotype definition. While this can be straight-forward for well-defined traits like height and BMI or disease conditions based on standardized ICD codes, it can be more challenging for phenotypes with a less structured definitions (e.g., behavioural traits) or that are characterized by a specific age of onset or dichotomized based on a (biomarker) threshold (e.g., obesity based on BMI). Finally, one should select a PGS with maximal SNP coverage in the target dataset; this is usually not a problem with high-density imputed genotypes or whole-genome sequencing, but can be limiting for samples genotyped on older arrays or with targeted genotyping/sequencing panels. After selecting a suitable PGS, it can be computed on the target set, e.g. via plink [43, 44]. To ensure a high quality, thorough quality control of the genotype data of the target set should be carried out including appropriate filters for minor allele frequency, genotype missing rate and Hardy-Weinberg-equilibrium. Challenges in the calculation mainly arise due to missing genotypes which can partly be replaced by proxy-SNPs or differences in the specification of the reference allele which can be re-coded.

Typically, the PGS of an individual cannot be interpreted directly but is compared to the distribution in a defined reference population. Additionally, different performance metrics should be taken into account. For quantitative phenotypes, the coefficient of determination *R^2^* is a common choice. It is often interpreted as the proportion of explained variance, however, its definition on test data is ambiguous [45]. Most often, it’s defined as the squared correlation of the PGS and the observed phenotype in the test set. As such, it can be interpreted as the proportion of explained variance on the test set when fitting a linear regression with the PGS as the only covariate. Noteworthy, it captures solely association of the PGS and the phenotype, not calibration which could be assessed via metrics such as the mean squared error of prediction or the mean absolute error of prediction. When interpreting *R^2^* is a common choice. It is often interpreted as the proportion of explained variance, however, its definition on test data is ambiguous [45]. Most often, it’s defined as the squared correlation of the PGS and the observed phenotype in the test set. As such, it can be interpreted as the proportion of explained variance on the test set when fitting a linear regression with the PGS as the only covariate. Noteworthy, it captures solely association of the PGS and the phenotype, not calibration which could be assessed via metrics such as the mean squared error of prediction or the mean absolute error of prediction. When interpreting *R^2^* values, it is crucial to take SNP-based heritability estimates as an upper bound into account. Most complex traits are not only influenced by polygenic signal but also by rare or structural variants as well as clinical and environmental factors. Methods like LD score regression provide estimates of the polygenic heritability of complex traits which is typically modest [46, 47]. Putting the *R^2^* in relation to this value increases interpretability. For qualitative outcomes, the *R^2^* can be replaced by Nagelkerke’s *R^2^* values, it is crucial to take snp-based heritability estimates as an upper bound into account. Most complex traits are not only influenced by polygenic signal but also by rare or structural variants as well as clinical and environmental factors. Methods like LD score regression provide estimates of the polygenic heritability of complex traits which is typically modest [46, 47]. Putting the *R^2^* in relation to this value increases interpretability. For qualitative outcomes, the *R^2^* can be replaced by Nagelkerke’s *R^2^* which measures the explained variability on a liability scale. Also the area under the receiver operator characteristic curve (AUC) is a common discriminatory measure for binary outcomes. For time-to-event data, this principle is represented via the C-index. For both, AUC and C-index, a value of 0.5 indicates no discriminatory power while a value of 1 refers to perfect discrimination of cases and controls. To take additionally calibration into account, the Brier score (binary) and integrated Brier score (time-to-event) are popular alternatives with lower scores indicating a better calibration and accuracy.

Due to the architecture of complex traits, in the subsequent analysis, PGS are typically integrated into (statistical) models alongside confounding factors (such as sex, age and genetic ancestry) as well as additional risk factors including environmental, lifestyle and clinical variables. Typically, the performance of PGS is then further assessed by incremental value, i.e. improvement of *R^2^*, *AUC* or C-index by the full model compared to a baseline model excluding the PGS. Furthermore, standardized effect estimates (*β*, odds ratio or hazard ratio per standard deviation) can be derived and quantify the polygenic component. Lastly, individuals are often grouped into PGS strata (e.g., based on PGS deciles) that enable more categorical comparisons (e.g., high PGS vs. medium PGS).

**Figure 2:**
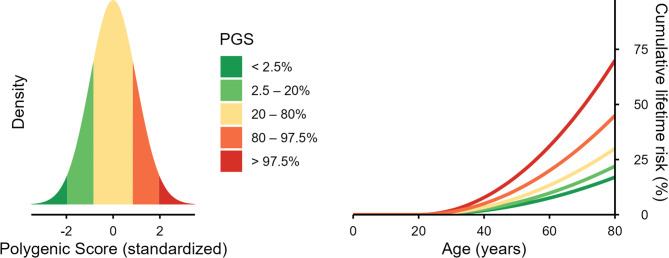
Risk stratification by PGS PGS-based stratification using quantiles of the standardized PGS distribution (left). Individuals are grouped into PGS strata (e.g., extreme low, intermediate, and extreme high percentiles), and the corresponding age-dependent cumulative disease risk is shown (right), highlighting increasing lifetime risk with higher PGS strata.

Among the first fields of application, polygenic contributions have been quantified in psychiatric diseases including schizophrenia and bipolar disorder [48]. Khera et al. showed that in some common and complex diseases, individuals with a high PGS show equivalent risk compared to individuals with a monogenic condition [49]. These studies gave insights into a new understanding of genetic predisposition and explained parts of observed heritability that could not be explained before [50 – 52]. Across studies, individuals in different strata of the PGS distribution show age-dependent differences in disease prevalence, supporting the use of PGS quantiles for risk stratification (Figure 2).

PGS studies are highly heterogeneous in both design and methodology, highlighting the need for standardized reporting to improve comparability [53]. A PGS report should clearly describe the study design, research objective, and outcome definition, as well as the study population, including recruitment strategy, demographic and clinical characteristics, and ancestral background. Any incorporated datasets, such as GWAS summary statistics, should be explicitly specified. Methodological reporting should include data pre-processing steps, including genetic quality control and handling of missing data. PGS development and evaluation should be conducted in independent samples. Accordingly, studies constructing a new PGS should detail cohort splitting procedures, the applied PGS method, and computational pipelines. For published scores, identifiers such as PMID or PGS Catalogue Score ID should be provided. Finally, reports should describe all statistical models, predictor variables, software, PGS distributions, and performance metrics relevant to the research aim. Limitations should be acknowledged, and code and, where possible, data should be made publicly available to ensure transparency and reproducibility.

## Translation of PGS into clinical practice

PGS can be translated into clinical care mainly as risk stratification tools rather than standalone prognostic markers. They estimate an individual’s relative genetic liability within a reference population and are clinically useful when combined with baseline risks and established predictors to guide threshold-based decisions on prevention, screening, or follow-up. The most advanced applications concern primarily the prevention of common multifactorial diseases [49]. For coronary artery disease and related cardiometabolic traits, integration of disease-specific PGS into established risk algorithms improves discrimination and, more importantly, reclassification of individuals at the borders of guideline-defined risk categories [54].

In oncology, PGS are increasingly embedded into multivariable models to enable risk-adapted screening. Breast cancer risk calculators such as BOADICEA/CanRisk now incorporate a PGS alongside classical risk factors and family history [55, 56]. Trial data from risk-stratified screening, such as WISDOM, further demonstrate that adding a PGS leads to clinically relevant changes in screening recommendations [57]. For prostate cancer, studies such as BARCODE1 have shown that targeting MRI and biopsy to men in the highest decile of a prostate cancer PGS yields a high detection rate of clinically significant tumours, including cancers that would not have fulfilled biopsy criteria under PSA- and MRI-based protocols alone [58].

A second clinical use case is refining risk in carriers of pathogenic variants in monogenic disease genes with incomplete penetrance. For breast and colorectal cancer, PGS can stratify carriers of moderate-penetrance variants such as CHEK2 or PMS2, with higher PGS strata approaching risks observed in carriers of established high-penetrance variants, such as BRCA1/2 or MLH1/MSH2, and lower strata closer to population-level risks [59, 60].PGS are also being explored as stratification tools in pharmacogenomics and therapeutic studies [61]. Post-hoc analyses of cardiovascular outcome trials have shown that patients with high polygenic risk for coronary artery disease derive greater absolute and relative benefit from intensive LDL-lowering therapy than those with low genetic risk, despite similar lipid reductions [62]. In parallel, genotype-based restrictions on single variants, such as APOE ε4 status for lecanemab in Alzheimer’s disease, illustrate how genetic markers can be tied directly to labelling and prescribing decisions [63].

Different implementation projects based on standardized electronic health record–linked care pathways, such as the eMERGE study, illustrate the integration of PGS into clinical workflows for common complex diseases, including coronary heart disease, cancer predisposition, and metabolic disorders [64]. In parallel, population programmes like Our Future Health plan to compute PGS for several diseases at scale and to investigate how these scores can inform risk-stratified prevention strategies [65].

Beyond classical clinical care settings, PGS have also been proposed for embryo selection in the context of in vitro fertilization, where embryos are ranked according to PGS for common diseases or even non-disease traits. At present, this use is highly controversial: for most traits, the proportion of variance explained by current PGS is modest and robust prospective validation is lacking. In addition, such applications raise substantial concerns about the potential for eugenic misuse and inequities in reproductive medicine, and are therefore viewed critically in current international ethical debates [66 – 69]. Nevertheless, several commercial providers, particularly in the US, have begun to implement PGS-based embryo selection, underlining the need for clear professional guidance and regulatory frameworks as polygenic scoring computation becomes technically straightforward and widely available [70].

Looking further ahead, conceptual discussions have also started to address the possibility of polygenic genome editing in embryos or germ cells. Simulation studies suggest that editing a limited number of variants could, in theory, produce relevant shifts in polygenic risk for several common diseases and related risk factors, with the potential to substantially reduce disease prevalence and to yield both individual-level benefits and reductions in health-care costs [71].

## Challenges and limitations

Despite new and larger data cohorts as well as continuous methodological progress (see previous section), there still remain substantial challenges:

Overall, the predictive performance of PGS remains limited [72]. For most complex traits, current models explain only a modest proportion of phenotypic variance, and their accuracy varies substantially across traits, cohorts, and ancestral backgrounds [73, 74]. Several factors contribute to this variability. Effect-size estimates used in PGS construction are typically derived from cohorts in populations of European ancestry, which limits transferability to other populations due to differences in LD structure, allele frequencies, and gene–environment interactions [73]. These issues have both methodological and ethical implications, as the application of PGS across ancestries may exacerbate disparities in predictive performance and clinical utility [74]. Thus, one of the main challenges at the current stage is the limited transferability of PGS across populations [75]. Potential solutions to this challenge come from two different angles. On the one hand, there is a need for more diverse cohorts and more cohorts with participants with other than European ancestry to develop population-specific scores [76]. On the other hand, also the methods for the development of PGS need to be adapted in order to yield also scores that are better to transfer or update when moving from one cohort to another and that can also work well with individuals with admixed ancestry [77, 78].

Importantly, PGS performance is influenced not only by ancestry differences, but also by additional sources of mismatch between the training dataset and the target population in which the score is applied. This applies, for example, to the translation of estimates from case-control studies to the population, where disease prevalence needs to be considered when interpreting genetic liability, heritability, and the expected strength of PGS-based risk stratification [79]. It is also relevant for population-scale applications, because many current and future scores are derived from large biobanks that often do not represent the general population. For example, the UK Biobank is a volunteer-based cohort recruited among middle-aged and older adults and is affected by healthy-volunteer bias, with participants being, on average, healthier, more educated, and socioeconomically less deprived than the underlying population [80]. This may also limit the capture of age-specific genetic effects, for instance when genetic factors play a larger role in early-onset phenotypes. More generally, participation-related biases may distort genetic associations and downstream estimates used for PGS development and evaluation.

Another key limitation is the incomplete capture of heritability by common variants [81–83]. Even the largest GWAS explain only part of the overall genetic contribution to complex traits, leaving a portion of “missing heritability” that may be attributable to rare variants, structural variation, gene-gene and gene-environment interactions, and regulatory mechanisms not adequately represented in current models [82, 84]. Recently, methodological developments have been made on the integration of rare variants [85], dominance effects [86] or gene-gene interactions [87]. Furthermore, environmental and lifestyle factors strongly modulate disease risk, and their interplay with genetic predisposition remains an active area of research [88–90]. Integrating environmental, epigenetic, and multi-omic information with genetic predictors is expected to improve both the predictive accuracy and biological interpretability of polygenic models [91, 92].

Finally, a key harmonization challenge arises from the inherently relative nature of PGS when they are implemented in clinical health-care systems that require standardized operating procedures (SOPs). By construction, a PGS is defined with respect to the allele frequency distribution, sequencing or genotyping platform, and imputation pipeline of the training population; consequently, the same numerical score does not necessarily correspond to the same absolute disease risk across cohorts or health-care systems. Robust clinical deployment therefore requires harmonized calibration frameworks that allow comparison of baseline hazards and enable systematic re-calibration when models are transferred to new datasets. In this context, large-scale, population-based national health-care datasets as well as clear reporting standards [53] may play a key role in supporting harmonization and cross-system comparability of PGS. In parallel, interoperable implementation pipelines and common data models are needed to support the integration of PGS with harmonized clinical, biomarker, and lifestyle data. Such infrastructures are essential to ensure that PGS-based decision support can be deployed consistently across centers, rather than resulting in locally defined and non-comparable scales of “genetic risk”.

## Conclusion and outlook

Recent years have seen substantial progress both regarding methodological advances for the development of PGS and substantial investments in population-based genetic cohorts. These developments should now help in the future to translate PGS from an academic concept towards routine clinical care. While there are first and promising developments in this direction [55, 60, 93], there are still some challenges ahead.

Moving forward will require not only more sophisticated methods that can take epistasis, non-linear (dominant) effects, interactions with environmental factors into account but also models that are better fine-mapped on the causal variants to enhance the transferability of PGS from one population to another. Beyond predictive discrimination, future work should increasingly focus on calibration, individual uncertainty quantification, and robust estimation across heterogeneous populations. An important aspect here could be the inclusion of even more advanced statistical modeling frameworks like distributional regression [94] or quantile regression [95, 96], that relate more aspects (beyond the mean [97]) of the distribution of the phenotype to the genetic variants. Lately, these methods have been also adapted to estimate PGS for the variance of a phenotype, which could even help to identify candidates for gene-environment interactions [98, 99]. Prospective cohorts also offer opportunities to develop longitudinal and time-to-event polygenic models that account for dynamic risk trajectories of the life course.

Additionally, there is a need for more diverse cohorts for PGS development with a focus on participants with non-European or admixed ancestry. To ensure that PGS are accepted both among patients and healthcare professionals, it is essential to make sure that the benefits are accessible also to populations currently underrepresented in genetic cohorts. In addition to predictive performance also under shifts of the distribution, interpretability of the models and transparent reporting will be critical for clinical acceptance and regulatory evaluation.

An important aspect of future research on translating PGS into clinical practice might be on how to include them in integrated clinical decision support systems, where the PGS for various phenotypes could be incorporated as baseline genetic liability that may complement or interact with variables from the clinical history or environment of the patient. These systems then need to focus on a clear clinical decision point, where the prediction really can help to guide potential preventive or therapeutic interventions. To assess the performance of these systems, there is a strong need for pragmatic clinical trials that help to externally validate the combination of different risk factors and PGS. This includes randomized controlled trials which can evaluate the benefit of PGS but are rarely conducted so far [57, 100].

To realize the full potential of PGS in clinical practice, interdisciplinary collaboration between clinical and methodological experts across disciplines is essential, alongside increasing genetic diversity in large-scale GWAS and biobanks to better represent population diversity and enable the development of more generalizable risk models. In parallel, technical best practices and reporting standards are needed to ensure portability, standardization, and reproducibility. Such standards will be essential for a reliable integration of PGS into clinical decision-making.
